# Age differences in brain signal variability are robust to multiple vascular controls

**DOI:** 10.1038/s41598-017-09752-7

**Published:** 2017-08-31

**Authors:** Douglas D. Garrett, Ulman Lindenberger, Richard D. Hoge, Claudine J. Gauthier

**Affiliations:** 1 Max Planck UCL Centre for Computational Psychiatry and Ageing Research, Berlin/London, Germany; 2 Center for Lifespan Psychology, Max Planck Institute for Human Development, Berlin, Germany; 3 European University Institute, San Domenico di Fiesole (FI), Fiesole, Italy; 4 Department of Neurology and Neurosurgery, McGill University, Montreal, Canada; 5 Department of Physics, Concordia University, Montreal, Canada; 6 PERFORM Centre, Concordia University, Montreal, Canada

## Abstract

A host of studies support that younger, better performing adults express greater moment-to-moment blood oxygen level-dependent (BOLD) signal variability (SD_BOLD_) in various cortical regions, supporting an emerging view that the aging brain may undergo a generalized reduction in dynamic range. However, the exact physiological nature of age differences in SD_BOLD_ remains understudied. In a sample of 29 younger and 45 older adults, we examined the contribution of vascular factors to age group differences in fixation-based SD_BOLD_ using (1) a dual-echo BOLD/pseudo-continuous arterial spin labeling (pCASL) sequence, and (2) hypercapnia via a computer-controlled gas delivery system. We tested the hypothesis that, although SD_BOLD_ may relate to individual differences in absolute cerebral blood flow (CBF), BOLD cerebrovascular reactivity (CVR), or maximum BOLD signal change (M), robust age differences in SD_BOLD_ would remain after multiple statistical controls for these vascular factors. As expected, our results demonstrated that brain regions in which younger adults expressed higher SD_BOLD_ persisted after comprehensive control of vascular effects. Our findings thus further establish BOLD signal variability as an important marker of the aging brain.

## Introduction

The study of lifespan development, cognition, and brain signal variability continues to gain momentum in cognitive neuroscience^1–7^ via EEG, MEG, and fMRI. In particular, a host of studies support that younger, better performing adults express greater moment-to-moment BOLD signal variability in various cortical regions^3, 8–12^. Overall, an emerging view states that brain signal variability may index a more effective, flexible system, and that the aging brain may undergo a generalized reduction in dynamic range^1, 13^.

However, the exact physiological nature of age differences in BOLD signal variability remains understudied. In particular, age differences in vascular properties could provide one potential reason why BOLD signal variability appears generally reduced in older adults^1, 13, 14^. Aging is known to be associated with hardening of blood vessel walls throughout the body^15, 16^; accordingly, increased rigidity in vessels of the brain could lead to a change in neurovascular coupling, with a decreased vascular response to a given level of metabolic demand^17^. Past attempts to address physiological confounds in BOLD variability studies involved the use of various proxy measures and techniques, such as manual and semi-automated independent component analysis (ICA)^18^ denoising pipelines, PHYCAA+^19^, and mixed-model control of level and change in observed blood pressure and heart rate^3, 8–10, 12^. However, more direct measures of vascular factors may be needed to support principled interpretations of age differences in BOLD signal variability. Specifically, it remains to be seen whether controlling for vascular rigidity and reactivity^20, 21^ would eliminate observed age group differences in BOLD signal variability.

Respiratory manipulations offer an excellent opportunity to examine this issue because such manipulations induce changes in BOLD signal via controlled vascular challenge. In particular, hypercapnia (i.e., breathing increased concentrations of CO_2_) leads to robust changes in cerebral blood flow (CBF) throughout gray matter via the vasodilatory properties of CO_2_^22, 23^. Hypercapnia yields substantial increases in BOLD signal throughout the brain that, in combination with quantification of the concomitant evoked change in CBF and end-tidal O_2_ concentrations, can be used to characterize the vascular component of the BOLD signal through a calibrated fMRI model^22, 24–28^. BOLD responses to hypercapnia can be used to estimate (1) BOLD cerebrovascular reactivity (CVR), defined as the increase in signal per unit of vasodilatory signal or mmHg CO_2_, and (2) the maximum possible BOLD signal change (M). CVR is thought to be an indicator of vascular health in the brain since it is a measure of vasodilatory capacity of brain blood vessels, and has been found to be reduced in stroke, carotid artery occlusion, and Alzheimer’s disease^17, 29–31^. M corresponds to the BOLD signal that would be obtained from complete elimination of deoxygenated hemoglobin from cerebral veins; it represents the dynamic range of the BOLD signal, and appears reduced in older adults^17, 32^. CBF, BOLD CVR, and M represent a comprehensive index of potential vascular contributions to BOLD, thus allowing us to address how accounting for such vascular factors may impact age group differences in BOLD signal variability. Although a host of studies have examined how various types of vascular scaling impact standard analyses of age differences in mean BOLD signals^17, 33, 34^, no study to date has examined whether typically found age differences in BOLD signal variability^1, 13^ remain robust after comprehensive control of vascular factors.

Accordingly, in a sample of younger and older adults scanned during fixation, we tested the hypothesis that although higher BOLD signal variability (SD_BOLD_fix)_ in younger adults may relate to CBF, BOLD CVR, or M (acquired via dual-echo BOLD/pseudo-continuous arterial spin labeling (pCASL) and hypercapnia), robust age group differences in BOLD variability would remain after multiple statistical controls for these vascular parameters. Given that BOLD CVR and M are typically (and in the present study) measured via hypercapnia during rest, we focus here only on fixation-based BOLD variability to better ensure that cognitive states under which all brain measures of interest are acquired (i.e., BOLD variability, CBF, BOLD CVR, and M) are comparable. Further, we also examine SD_BOLD_fix_ within data that have been carefully denoised prior to estimation of age differences or vascular effects; as such, only relatively artifact-free SD_BOLD_fix_ data are analyzed in our models of interest.

## Methods

### Participants

Acquisitions were originally conducted in 31 young (10 females, 19–32 yrs, mean age = 23.74 ± 2.90 yrs) and 51 older (34 female, 55–72 yrs, mean age = 63.18 ± 4.82 yrs) healthy participants on a Siemens TIM Trio 3 T magnetic resonance imaging (MRI) system (Siemens Medical Solutions, Erlangen, Germany) using the vendor-supplied 32-channel receive-only head coil for all acquisitions (see ref. 17). All participants gave informed consent, the local ethics committee (Comité mixte d’éthique de la recherche du Regroupement Neuroimagerie/Québec) approved the study, and all methods were performed in accordance with the relevant approved guidelines and regulations. See further details below regarding how the final sample (*n* = 74, 29 young adults (9 females), 45 older adults (32 females)) was determined in the context of the current study.

Exclusion criteria for this study included claustrophobia, cardiac disease, hypertension or taking medication to lower blood pressure, neurological or psychiatric illness, smoking, excessive drinking (more than 2 drinks per day), thyroid disease, diabetes, asthma, and using a regular treatment known to be vasoactive or psychoactive. Participants were all nonsmokers, or had been nonsmokers for at least 5 years. Older participants met with a geriatric MD to ensure that they did not meet any of the exclusion criteria for the study. All participants completed a short neuropsychological screening battery to assess normal cognition. The cognitive characterization of this cohort has been published before and results can be found in Table 1 of Gauthier *et al*.^17^. Older adults participants also completed the Mini-Mental State Examination^35^ to screen for global cognitive decline; no participant scored less than 26.

**Table 1:** Multivariate PLS model peak activations, bootstrap ratios, and cluster sizes for regions showing increased BOLD signal variability with age.

Region	Hem	MNI coordinates			BSR	Cluster size (voxels)
		X	Y	Z		
Precentral gyrus	R	48	8	44	4.87	49
Precuneus	L	−4	−64	32	4.86	167
Anterior cingulate	L	0	32	24	3.90	45
Superior medial gyrus	L	0	52	8	3.64	20
Middle temporal gyrus	R	56	−48	12	3.61	18
Middle occipital gyrus	L	−44	−76	36	3.39	16
Angular gyrus	L	−56	−68	24	2.83	16

Note: SD = standard deviation; BOLD = blood oxygen level-dependent; Hem = hemisphere; MNI = Montreal Neurological Institute; BSR = bootstrap ratio (model salience/bootstrapped standard error).

Whenever possible, participants needing eyesight correction were asked to wear contact lenses on the day of the MRI experiment. For those without contact lenses, eyesight was corrected to the nearest possible 0.50 D using MRI-compatible glasses. For participants with significant hearing losses, written instructions were projected onto a screen at the end of the bore that could be seen by participants through a mirror.

### MRI data

The current paper examines rest block data from a previously published block design task paradigm^17^. In total, there were five 60 sec rest blocks available for analysis in the current study (total = 300 sec). Rest blocks from this paradigm were of focus given that hypercapnia measures were also collected during resting-state (see below), thus matching the cognitive states of BOLD and vascular data as closely as possible.

### Magnetic resonance image acquisition

Sessions included two anatomical, 1 × 1 × 1 mm magnetization prepared rapid gradient echo (MPRAGE) acquisitions with repetition time (TR)/echo time (TE)/flip angle = 2300 ms/3 ms/9 deg, 256 × 240 matrix, and a generalized autocalibrating partially parallel acquisition (GRAPPA) acceleration factor of two^36^. Older participants had an additional fluid attenuated inversion recovery (FLAIR) acquisition to estimate the presence and severity of white matter lesions. FLAIR acquisition parameters included TR/TE/flip angle = 9000 ms/107 ms/120 with echo train length of 15, an inversion time of 2500 ms, 512 × 512 matrix for an in-plane resolution of 0.43 × 0.43 mm and 25 slices of 4.8 mm. White matter hyperintensities were quantified using the scale from Wahlund *et al*.^37^. Only participants with scores of 0 or 1, corresponding to no or few small lesions, were included in this study. The score average and standard deviation was 0.67 ± 0.48 in the older group.

Functional image series were acquired using a dual-echo pseudo-continuous arterial spin labeling acquisition^38^ to measure changes in CBF and BOLD signal simultaneously. The parameters used include: TR = 3000 ms, TE #1 = 10 ms, TE #2 = 30 ms, flip angle = 90 deg, with 4 × 4 mm in-plane resolution and 11 slices of 7 mm (1 mm slice gap) on a 64 × 64 matrix (at 7/8 partial Fourier). GRAPPA acceleration factor = 2, postlabeling delay = 900 ms, label offset = 100 mm, Hanning window-shaped right frontal pulse with duration/space = 500 ms/360 ms, flip angle of labeling pulse = 25 deg, slice-selective gradient = 6 mT/m, tagging duration = 1.5 seconds^38^.

We conducted two imaging sub-sessions within the same overall session. In the first sub-session, MPRAGE, FLAIR, and fMRI data were acquired. In the second sub-session, MPRAGE and the hypercapnia functional acquisition were performed. Participants were taken out of the scanner in between these two acquisition segments to either put on, or take off the hypercapnia mask, depending on the order of acquisitions. Participants were allowed to move or stretch during this pause to ensure greater comfort, especially in the older participants.

### Hypercapnic manipulation

Hypercapnic manipulations were achieved using a computer-controlled gas delivery system in combination with a sequential gas delivery circuit (RespirAct system; Thornhill Research Inc, Toronto, Ontario, Canada). The RespirAct system allows independent control of end-tidal partial pressure of CO_2_ (PCO_2_) and end-tidal partial pressure of O_2_ (PO_2_) using a feed-forward physiological model, using as input the measured or predicted baseline O_2_ consumption and CO_2_ production of a subject^39^. PCO_2_ was targeted to be 40 mm Hg at baseline and 45 mm Hg during the hypercapnia blocks. These values were maintained throughout each block. PO_2_ was targeted to be 100 mm Hg throughout the experiment. Gas was sampled continuously (via RespirAct) at the mouth and analyzed for PCO_2_ and PO_2_. During the hypercapnic stimulation, volunteers breathed through the circuit via a soft plastic mask sealed to the face using adhesive dressing (Tegaderm 3 M Healthcare, St. Paul, MN, USA), as necessary to prevent gas leakage. Participants were asked to breathe deeply enough to empty the fresh gas compartment of the breathing circuit at every breath during the functional acquisitions (to ensure delivery of the entire gas dose delivered by the machine and stable end-tidal values throughout the block). Generally, subjects did not have difficulty complying with this requirement.

Participants underwent the manipulation twice during the study, once outside the scanner before the imaging session for acclimation, and once during the MRI session. Subjects were interviewed after the acclimation session to assess their level of respiratory discomfort using the 7-point scale published by Banzett *et al*.^40^. Subjects reporting a subjective rating of 5 or greater (moderate discomfort or greater) were not invited to continue in the study (2 cases). The average discomfort rating over all subjects was 2.22 ± 1.03, corresponding to only slight discomfort that could be maintained for long periods.

### Data preprocessing

fMRI and ASL data were preprocessed with FSL 5^41, 42^ and Neurolens (www.neurolens.org). Pre-processing included: motion-correction with spatial smoothing (8 mm full-width at half maximum Gaussian kernel) and high-pass filtering (0.01 Hz). The CBF signal was isolated from the series of first echoes using linear surround subtraction^43^, and the BOLD signal was extracted using linear surround addition of the second echo series^43–45^ in Neurolens. Registration of TE = 30 ms (i.e., BOLD) functional images to high-resolution participant-specific T1 images, and from T1 to 2 mm standard space (MNI 152_T1) was carried out using FLIRT. These same spatial transformation matrices were then used to register the TE = 10 ms (i.e., ASL) data, to minimize normalization errors due to bright scalp signal typical of ASL images.

Beyond standard preprocessing steps, we subsequently examined all functional volumes for artifacts via independent component analysis (ICA) within-run, within-person, as implemented in FSL/MELODIC^18^. Noise components were targeted according to several key criteria: (a) Spiking (components dominated by abrupt time series spikes ≥6 SDs); (b) Motion (prominent edge or “ringing” effects, sometimes [but not always] accompanied by large time series spikes); (c) Susceptibility and flow artifacts (prominent air-tissue boundary or sinus activation; typically represents cardio/respiratory effects); (d) White matter (WM) and ventricle activation^46^; (e) Low-frequency signal drift^47^; (f) High power in high-frequency ranges unlikely to represent neural activity (≥75% of total spectral power present above 0.13 Hz;); and (g) Spatial distribution (“spotty” or “speckled” spatial pattern that appears scattered randomly across ≥25% of the brain, with few if any clusters with ≥10 contiguous voxels [at 4 × 4 × 4 mm voxel size]). Examples of these various components we typically deem to be noise can be found in supplementary materials in Garrett *et al*.^48^. By default, we utilize a conservative set of rejection criteria; if manual classification decisions are difficult due to the co-occurrence of apparent “signal” and “noise” in a single component, we typically elect to keep such components, which helps guard against potential concerns that ICA denoising may remove “signal” of interest^49^. Two independent raters of noise components were utilized, and >90% inter-rater reliability was required on separate data before denoising decisions were made on the current data. Components identified as artifacts were then regressed from corresponding fMRI runs using the FSL regfilt command. The use of ICA denoising had dramatic effects in our past research, effectively doubling the predictive power of BOLD signal variability^8^. Thus, calculating BOLD signal variance from relatively artifact-free BOLD time series permits the examination of what is more likely meaningful brain signal dynamics. Furthermore, as the current study seeks to identify possible vascular biases in existing methods, we elected to preprocess the data using the same techniques as in previously published studies^3, 8, 12, 50^.

The pre-processed flow-weighted time series from the 10 ms TE (short echo) was also denoised using the same process to ensure comparable removal of motion and other artifact components (e.g., drifts, spikes). All the same exclusion criteria were used for these datasets, and two additional rejection criteria were added to take into account ASL-specific artifacts, likely due to tag and fat saturation instabilities. The components presumably due to tag effects took the form of large areas of highly positively or negatively correlated signals. These areas did not follow anatomical boundaries and were typically largest in the bottom slices of the volume. The fat saturation components were a crescent-shaped area of the same shape as the back of the head shifted into occipital areas.

### Computation of SD_BOLD_ during fixation blocks (SD_BOLD_fix_)

One of several BOLD signal variance measures utilized in fMRI research (e.g., amplitude of low frequency fluctuations, or ALFF^51^), we focused on a modified voxel-wise time series standard deviation from fixation block data (SD_BOLD_fix_). SD_BOLD_ does not require continuous data, making it an effective measure of signal variation in concatenated (discontinuous) block data such as those utilized in the current study. We do not employ Fourier-based measures such as ALFF in the present study explicitly due to our use of concatenated (discontinuous) block data. The validity of frequency-specific power estimates taken via ALFF relies on continuous time. Due to the concatenated (discontinuous) block nature of our data, only local within-block data would be amenable to frequency-specific power estimation in our study (30 secs per block; using a rule of thumb minimum 3 cycles of any estimable frequency = only frequencies ≥0.10 Hz are estimable), thus precluding estimation of power within a typical bandpass range in BOLD data (0.01 to 0.10 Hz).

To compute SD_BOLD_fix_ from the current fixation block data, we first performed a block-normalization procedure to account for residual low-frequency artifacts. We normalized all fixation blocks such that the overall 4D mean (x*y*z*time) across brain and block was 100. For each voxel, we then subtracted the block mean and concatenated across all blocks. Finally, we calculated voxel standard deviations across this concatenated time series^8–10, 48^. Our computation of SD_BOLD_ differs from resting-state-fluctuation analysis (RSFA^52^, another time series SD estimation method) in that the original RSFA does not normalize for 4D means, nor blocks, in the same manner. So-called “normalized” RSFA^33^ does, however, remove the entire time-series mean prior to SD calculation, but this is not relevant for a block design such as ours as we do not consider meaningful the differences between discontinuously acquired blocks, given that extreme mean differences between blocks can dramatically inflate SD estimates^8, 9^. Critically, regardless of the extent that SD_BOLD_fix_ and RSFA may relate mathematically, our examination of SD_BOLD_ occurs only after extensive multi-stage data denoising routines. Accordingly, for the remainder of the current paper, our use of the terms “SD_BOLD_fix_” or “BOLD signal variability” thus refer to signal variability resulting from already cleaned, denoised, relatively artifact-free, and multiply normalized time series data.

As noted above, the fixation blocks we analyzed in the current study are extracted from an alternating block design (fixation-task-fixation-task). We chose to examine only fixation blocks in the current study to maintain maximum overlap in “brain or cognitive state” between BOLD, and baseline CBF and hypercapnia data. Hypercapnia data are typically, as in the current study, collected only off-task. This ensures that any comparison of age prediction by both SD_BOLD_fix_ and vascular measures are not confounded by presumed differences in cognitive state.

However, one potential concern regarding SD_BOLD_fix_ in the present study may be that because fixation blocks alternate between task blocks, it is possible that the temporal variability in any given fixation block may be impacted by the signal dynamics of the immediately preceding task block. If so, a preceding block should have the greatest impact on variance within the first portion of the succeeding block (i.e., due to BOLD signal (or brain state) spillover from the preceding block), rather than within later portions of the succeeding block. However, we showed extensively in past work^10^ that the split-half reliability of SD_BOLD_fix_ values from concatenated first and concatenated second block halves for fixation block-based SD_BOLD_fix_ estimation was near unity in younger and older adult groups (each *r* = ~0.97). This suggests that estimation of SD_BOLD_ from fixation blocks is robust despite embedding within a broader alternating block-design study. We thus apply the same logic in the current study when linking SD_BOLD_fix_ to vascular measures obtained during resting periods.

### Vascular parameter estimation

Baseline CBF, CVR and M values were obtained using Neurolens and in-house Matlab code (for M). These vascular and metabolic parameters were selected for the current study since they have been previously shown to robustly capture several important aspects of age-related differences in hemodynamics^21, 44, 53–55^. Fractional changes in BOLD and CBF signals were then determined for hypercapnia by fitting a general linear model (GLM) to the respective signals and dividing the estimated effect size by the estimated constant term. Model fits used a single-gamma hemodynamic response function with parameters described by Glover^56^ and included linear, quadratic, and cubic polynomials to represent baseline signal and drifts.

Absolute resting CBF was determined from the pseudocontinuous arterial spin labeling data using the approach described by Wang *et al*.,^57^ assuming blood-brain partition coefficient = 0.9, labeling efficiency = 0.80, blood T1 = 1.49 seconds, and gray matter T1 = 1.4 seconds. For this computation, the baseline ASL difference signal estimated in the GLM fit for each gas manipulation was divided by the corresponding unsubtracted baseline EPI signal from the ASL series, computed in a similar GLM fit carried out on the unsubtracted EPI series. The unsubtracted baseline EPI signal from the ASL series is used here as a surrogate for the fully relaxed magnetization that can alternately be acquired in the form of what is termed an M_0_ scan. To account for incomplete recovery of longitudinal magnetization during the sequence TR of 3 seconds, baseline EPI estimates from gray matter ROIs were corrected using the gray matter T1 value cited above. The resultant ratio was converted to absolute CBF units based on the parameters above. CVR was obtained by dividing the percent BOLD signal changes during the hypercapnia manipulation by the increase in end-tidal PCO_2_ values during this manipulation^44, 58, 59^. BOLD CVR was used rather than CBF CVR since BOLD CVR was shown earlier to be a more sensitive measure of vascular change in aging^17^.

1 $$\begin{array}{c}CVR=\,\frac{ \% {\rm{\Delta }}BOL{D}_{C{O}_{2}}}{{\rm{\Delta }}PC{O}_{2}}\end{array}$$

The generalized calibration model (GCM) method^28, 44^ was utilized to obtain M estimates for each subject, using ROI-averaged values for relative CBF and BOLD signal changes during hypercapnia.

2 $$\begin{array}{c}M=\frac{\frac{{\rm{\Delta }}BOLD}{BOL{D}_{0}}}{1-{(\frac{CBF}{CB{F}_{0}})}^{\alpha }-\,{(\frac{(1-S{V}_{{O}_{2}})}{({1-S{V}_{{O}_{2}}|}_{0})})}^{\beta }}\end{array}$$

where α is the flow-volume coupling during hypercapnia and is assumed to have a value of 0.18^60^ and β is the influence of deoxygenated hemoglobin on transverse relaxation^61^. A β value of 1.5 was used here. SV_O2_ is the venous oxygen saturation and is calculated from end-tidal O_2_ concentrations (PO_2_) and CBF using a series of equations described previously^28, 44^.

### Multivariate statistical analyses and determination of regions for brain parameter estimation

To examine multivariate relations between SD_BOLD_fix_ and age group during rest blocks, we employed a “Task PLS” analysis^62, 63^. Task PLS begins by calculating a between-subject covariance matrix (COV) between experimental conditions/groups and each voxel’s SD_BOLD_fix_. COV is then decomposed using singular value decomposition (SVD).

3 $$\begin{array}{c}SV{D}_{COV}=USV^{\prime} \end{array}$$

This decomposition produces a left singular vector of experimental condition/group weights (*U*), a right singular vector of brain voxel weights (*V*), and a diagonal matrix of singular values (*S*). This analysis produces orthogonal latent variables (LVs) that optimally represent relations between experimental conditions/groups and voxel-wise SD_BOLD_ values. Each LV contains a spatial activity pattern depicting the brain regions that show the strongest relation to condition/group contrasts identified by the LV. Each voxel weight (in *V*) is proportional to the covariance between voxel SD_BOLD_ and the condition/group contrast. In the current study, only one LV was estimable, given that we examined a single condition (fixation) across two age groups (i.e., a single PLS-derived contrast captures the associated age group effect on SD_BOLD_).

Significance of detected relations between multivariate spatial patterns and conditions/groups was assessed using 1000 permutation tests of the singular value corresponding to each LV. A subsequent bootstrapping procedure revealed the robustness of voxel saliences across 1000 bootstrapped resamples of the data^64^. By dividing each voxel’s mean salience by its bootstrapped standard error, we obtained “bootstrap ratios” (BSRs) as normalized estimates of robustness. We thresholded BSRs at conservative values of ±2.70, which exceeds a 99% confidence interval.

Typically, to obtain a PLS-based summary measure of each participant’s robust expression of a particular LV’s spatial pattern, one would calculate within-person “brain scores” by multiplying each voxel (*i*)’s weight (*V*) from each LV (*j*) (produced from the SVD in equation (1)) by voxel (*i*)’s SD_BOLD_ value, for each condition/group (*k*) within person (*l*), and summing over all (*n*) brain voxels:

4 $$\sum _{i=1}^{n}{V}_{ij}S{D}_{BOL{D}_{ikl}}$$

This is equivalent to the vector multiplication of V by a subject’s vector of SD_BOLD_ values for all voxels. However, in the current study, to test the impact of vascular effects on age differences in SD_BOLD_fix_, we wanted to constrain all subsequent analyses only to brain scores calculated from regions thresholded by bootstrapping procedures that expressed higher SD_BOLD_fix_ in younger adults. We then utilized these same thresholded regions for extracting single, averaged vascular parameters (absolute CBF, BOLD CVR, and M). This allowed us to address in a spatially specific manner whether higher SD_BOLD_fix_ typically seen in younger vs. older adults can be accounted for by vascular factors.

To restrict all multivariate analyses to grey matter (GM) from the denoised images (which were analyzed whole brain), we masked our functional data with the GM tissue prior provided in FSL (resampled to 4 mm). We localized thresholded peaks from PLS model output by submitting resulting MNI coordinates to the Anatomy Toolbox (version 1.8) in SPM8, which applies probabilistic algorithms to determine the cytoarchitectonic labeling of MNI coordinates^65, 66^.

### Final regression models

Given our primary goal to test whether PLS-derived age differences in SD_BOLD_fix_ could be accounted for by vascular factors, we fit a series of subsequent regression models in which age group and either one or all of the vascular predictors (absolute baseline CBF, BOLD CVR, and M) predicted SD_BOLD_fix_. Finally, we evaluated results using 1000 bootstrap resamples (with replacement) of the data.

### Treatment of distributions, and subsequent univariate and multivariate outlier detection

**Figure 1:**
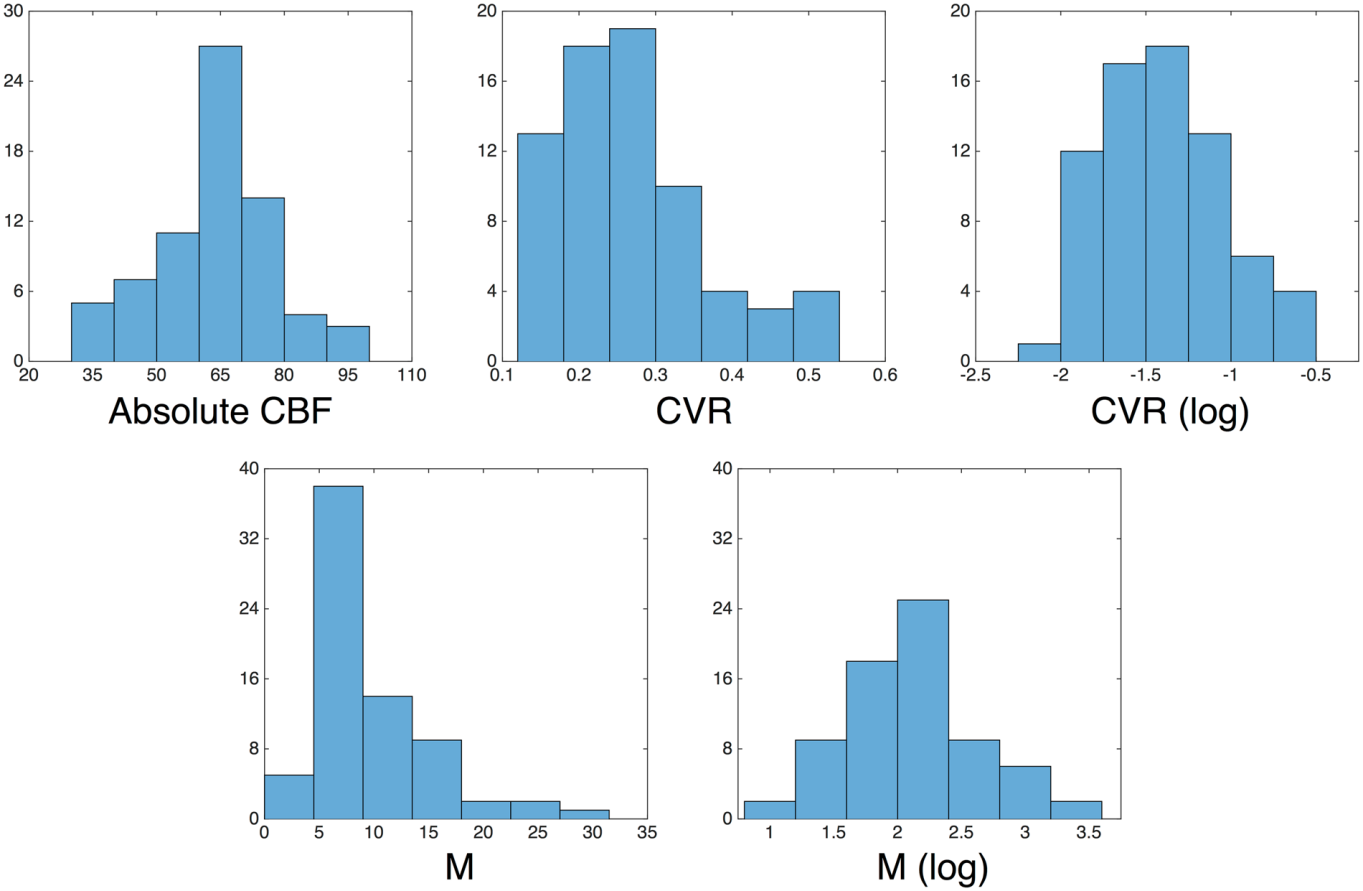
Histograms of absolute CBF, and pre- and post-transformed CVR and M values utilized in Models 1–4. Note: CBF = cerebral blood flow; CVR = cerebrovascular reactivity; M = maximal BOLD signal change.

Inspection of distributions occurred for all variables prior to all regression model runs. Univariate outlier detection was performed on all measures prior to regression modeling noted above; cases that were greater than ± 2.5 SDs from sample means on any variable were removed. The removal of univariate outlier cases yielded an effective sample size of *n* = 74 (29 young, 45 older) prior to multivariate outlier detection (see below). Following this step, BOLD CVR and M distributions still deviated from Gaussian normality (1-sample Kolmogorov-Smirnov Test, *both ps* < 0.05) and were both right skewed. We thus log transformed the BOLD CVR and M variables, which produced Gaussian distributions for both (1-sample Kolmogorov-Smirnov Test, both *ps* > 0.10). CBF, and pre- and post-transformed CVR and M distributions, are noted in Fig. 1).

Multivariate normality was then assessed for the full regression model run (Table 2, Model 4) by estimating the Mahalanobis distance (*D* ^2^) for each subject. As Mahalanobis distances are approximately Χ^2^ distributed, we compared our *D* ^2^ values (*df* = 4, where *df* represents the number of predictors in the model (age group, absolute CBF, BOLD CVR, and M)) to a reference Χ^2^ distribution within a Q-Q plot. Extreme values in the tail of the *D* ^2^ distribution that departed from the reference distribution were removed, while maintaining that no more than ~5% of the model cases be held out, no matter how extreme the absolute *D*_2_ value; in our model, this amounted to a reduction of three cases out of 74 (final *n* = 71; 28 young, 43 older adults). To maintain comparability of samples, all sub-models (Table 2, Models 1–3) were run using the same *n* = 71 that remained after multivariate outlier targeting for the full model. All regression models and outlier detection steps were run using SPSS 23 (IBM, Inc.).

**Table 2:** Univariate models.

Model	Dependent variable	Predictor	*b*	Bootstrap 95% CI	*se*	*t*	*p*	Zero- order	Partial	Semi- Partial	VIF
1	SD_BOLD_ brain score	Age Group	−0.89	(−1.16, −0.59)	0.14	−6.21	**3**.67 * 10 ^−8^	−0.61	−0.60	−0.60	1.09
		Absolute CBF	0.00	(−0.01, 0.01)	0.01	−0.44	0.66	0.13	−0.05	−0.04	1.09
2	SD_BOLD_ brain score	Age Group	−0.69	(−1.09, −0.34)	0.17	−3.97	**1**.77 * 10 ^−4^	−0.61	−0.43	−0.37	1.66
		BOLD CVR	0.40	(−0.08, 0.80)	0.24	1.66	0.10	0.51	0.20	0.16	1.66
3	SD_BOLD_ brain score	Age Group	−0.75	(−1.06, −0.43)	0.16	−4.73	**1**.16 * 10 ^−5^	−0.61	−0.50	−0.45	1.37
		M	0.24	(−0.12, 0.55)	0.16	1.49	0.14	0.44	0.18	0.14	1.37
4	SD_BOLD_ brain score	Age Group	−0.67	(−1.03, −0.031)	0.19	−3.57	**6**.79 * 10 ^−4^	−0.61	−0.40	−0.34	1.90
		Absolute CBF	0.00	(−0.01, 0.01)	0.01	−0.39	0.70	0.13	−0.05	−0.04	1.15
		BOLD CVR	0.29	(−0.21. 0.80)	0.27	1.08	0.28	0.51	0.13	0.10	2.01
		M	0.17	(−0.24, 0.53)	0.17	0.99	0.33	0.44	0.12	0.09	1.64
5	Corr_SD−CBF_	Age Group	−0.05	(−0.12, 0.02)	0.04	−1.30	0.20	−0.16	n/a	n/a	n/a
6	Corr_SD−CVR_	Age Group	−0.10	(−0.17, −0.02)	0.04	−2.50	**0**.**02**	−0.29	n/a	n/a	n/a

Note: SD = standard deviation; CI = confidence interval; BOLD = blood oxygen level-dependent; YA = young adults; OA = older adults; CBF = cerebral blood flow; CVR = cerebrovascular reactivity; M = maximal BOLD signal change; VIF = variance inflation factor. Significant *p*-values are in bold font. “Zero-order”, “partial,” and “semi-partial” columns reflect effect sizes in Pearson’s correlation metric. Levene’s test for equality of variances was insignificant in all models (*p*s ranged from 0.12 to 0.95). Finally, the frequency of males and females was significantly different in the young (20 male, 8 female) and older (11 male, 32 female) groups (Chi square = 14.49, *p* = 1.41 * 10^−4^); however, sex had no predictive effect in any model reported above (all *p*s ranged from 0.21 to 0.52), and also had no appreciable effect on the unique effect of any predictor of interest. We thus report all final models above without further control for participant sex. Finally, there were no robust interactions between age group and any vascular parameter in Models 1–4 (all *p*s > 0.25).

## Results

### PLS results

**Figure 2:**
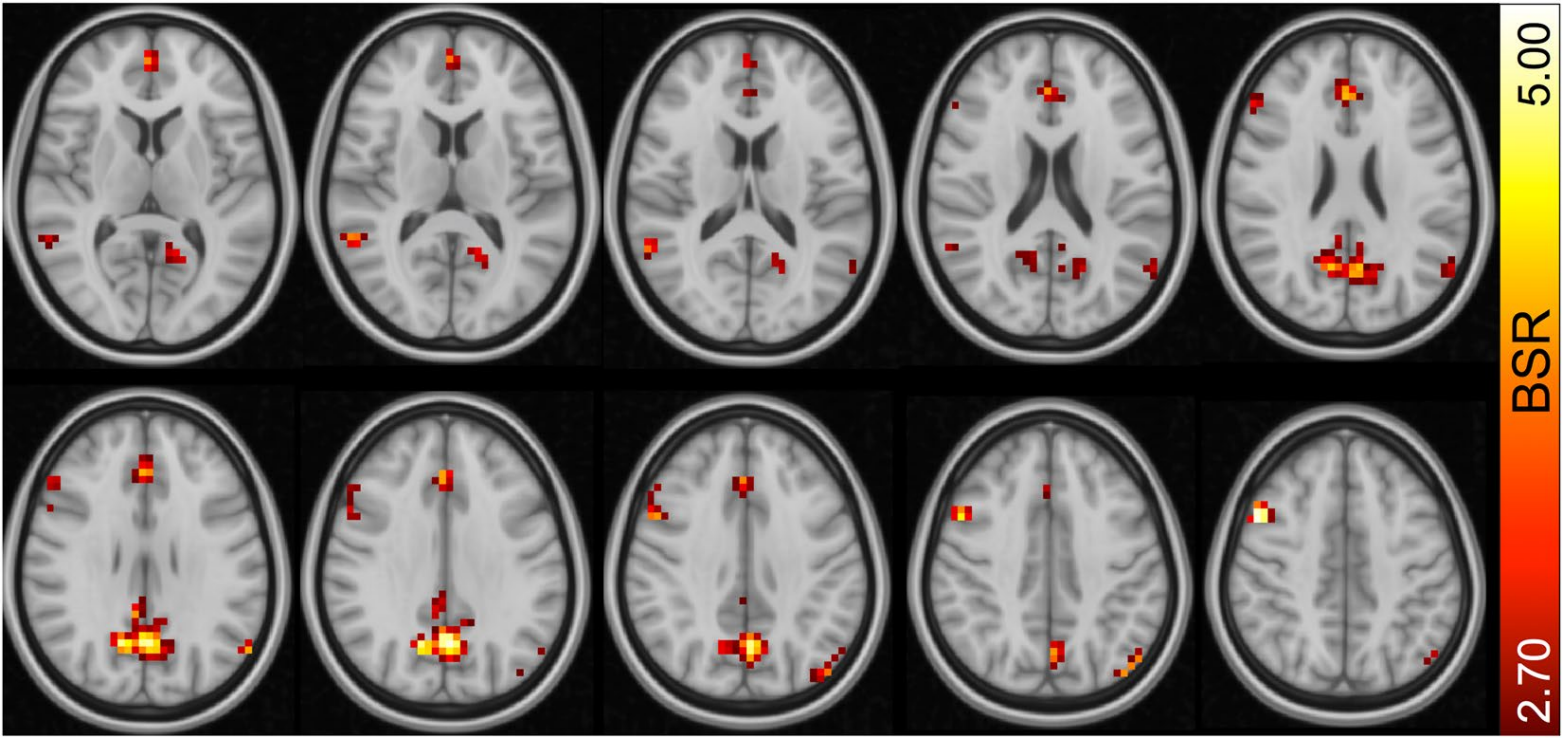
Regions expressing greater SD_BOLD_ in younger vs. older adults. Note: BSR = bootstrap ratio. From top left, slices are shown from Z = 8 to Z = 44 in 4 mm increments.

We first ran a multivariate partial least squares (PLS) model (see Methods for details) to test for regions in which younger adults expressed greater SD_BOLD_fix_, and we found a single robust latent variable (permuted *p* = 0.012) representing this relationship. The thresholded brain pattern (Fig. 2) highlights regions in which younger adults (YA) expressed higher SD_BOLD_fix_. This model revealed several regional effects that are in general accordance with previous studies. In particular, precuneus, anterior cingulate, and DLPFC often exhibit higher SD_BOLD_fix_ in younger adults^7–10, 67^. These convergent findings position the current dataset well for subsequent examination of whether the effect of significantly higher SD_BOLD_fix_ in younger adults can be eliminated by accounting for vascular factors. A complete list of bootstrapped cluster peaks can be found in Table 1.

### Regression models linking age and vascular measures to SD_BOLD_fix_

Next, we extracted baseline CBF, BOLD CVR, and M from the regions showing higher SD_BOLD_fix_ levels in younger adults in the PLS model noted above. We then fit several models using 1000 resamples (with replacement) of the data, regressing SD_BOLD_-based brain scores (from the age-based PLS model above) on age group and the various vascular measures. This allowed us to test whether initial age differences in SD_BOLD_ could be attributed to vascular factors. We began by running separate models with age group and each vascular predictor (to give each vascular predictor a maximal chance to account for variance in SD_BOLD_fix_), and then a full model with age group and all vascular predictors together. As hypothesized, our results indicated that age differences in SD_BOLD_fix_ remained regardless of whether age was pitted against single or against multiple vascular predictors (Table 2, Models 1–4). BOLD CVR (*r* = 0.51) and M (*r* = 0.44) indeed expressed moderate zero-order relations to SD_BOLD_ (but only weakly for CBF, *r* = 0.13), although this initial predictive utility for vascular parameters was largely eliminated when controlling for age group (unique (semi-partial) *r*s = −0.04 (CBF), 0.16 (BOLD CVR), and 0.14 (M)). However, this in turn indicates that a certain amount of predictive variance attributed to vascular parameters was shared with age group, reflected in differential reductions in the zero-order predictive utility of age (*r* = −0.61) across models (semi-partial *r*s; Model 1 = −0.60; Model 2 = −0.37; Model 3 = −0.45; Model 4 = −0.34) in Table 2.

### Distributional analyses of links between SD_BOLD_fix_ and CVR/CBF

**Figure 3:**
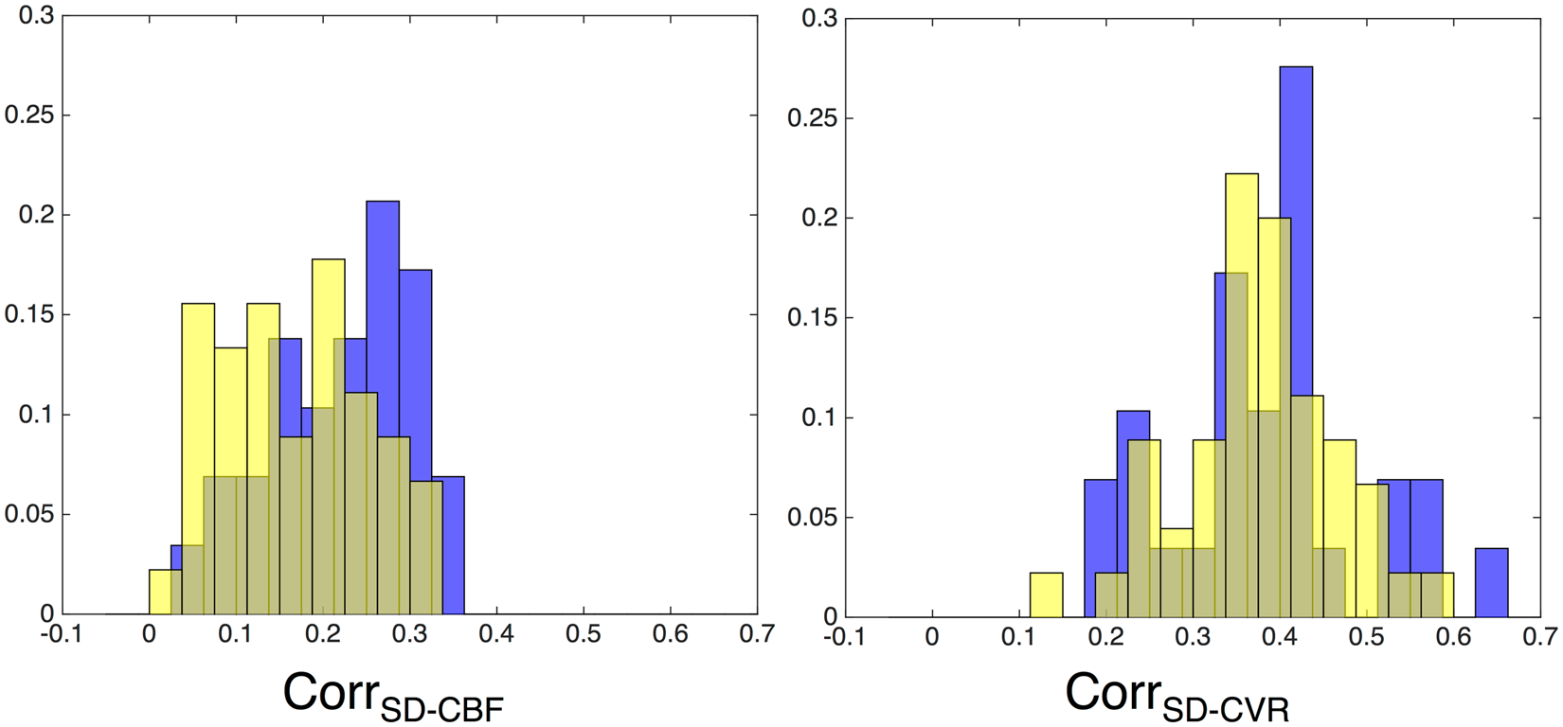
Histograms of within-subject voxel-wise correlations of SD_BOLD_-CBF and SD_BOLD_-CVR relations for younger (blue) and older (yellow) adults. Note: Y-axis values represent normalized histogram proportions due to different sample sizes in each age group (young adults, *n* = 29; older adults *n* = 42).

Next, we examined the within-person correlations between SD_BOLD_fix_ and CBF, and SD_BOLD_fix_ and CVR, across all voxels in the brain, to identify the range of relations between SD_BOLD_fix_ and vascular measures across persons and age groups. While very recent improvements in ASL acquisitions and analysis also make voxel-wise M estimation more feasible, the current data only allow reliable M estimation through averaging across multiple voxels in an ROI^17^. Resulting distributions can be found in Fig. 3. We find that there is not only a remarkably wide range in correlation between SD_BOLD_fix_ and vascular measures across subjects, but also minimal (Table 2, Model 5) or modest (Table 2, Model 6) effect sizes representing differences between age groups in these values. This suggests that on average, age groups are not dramatically different in how SD_BOLD_fix_ and vascular parameters relate across the brain, and that the *strength* of these relations varies widely across persons. These findings have direct relevance to future approaches seeking voxel-wise, within-subject calibration of fMRI signal variance. The logic of within-person calibration relies on the presumed reliability of relationship between BOLD and vascular measures. Recent work may however make it possible to obtain more robust calibrated fMRI estimates in the future^68, 69^.

**Figure 4:**
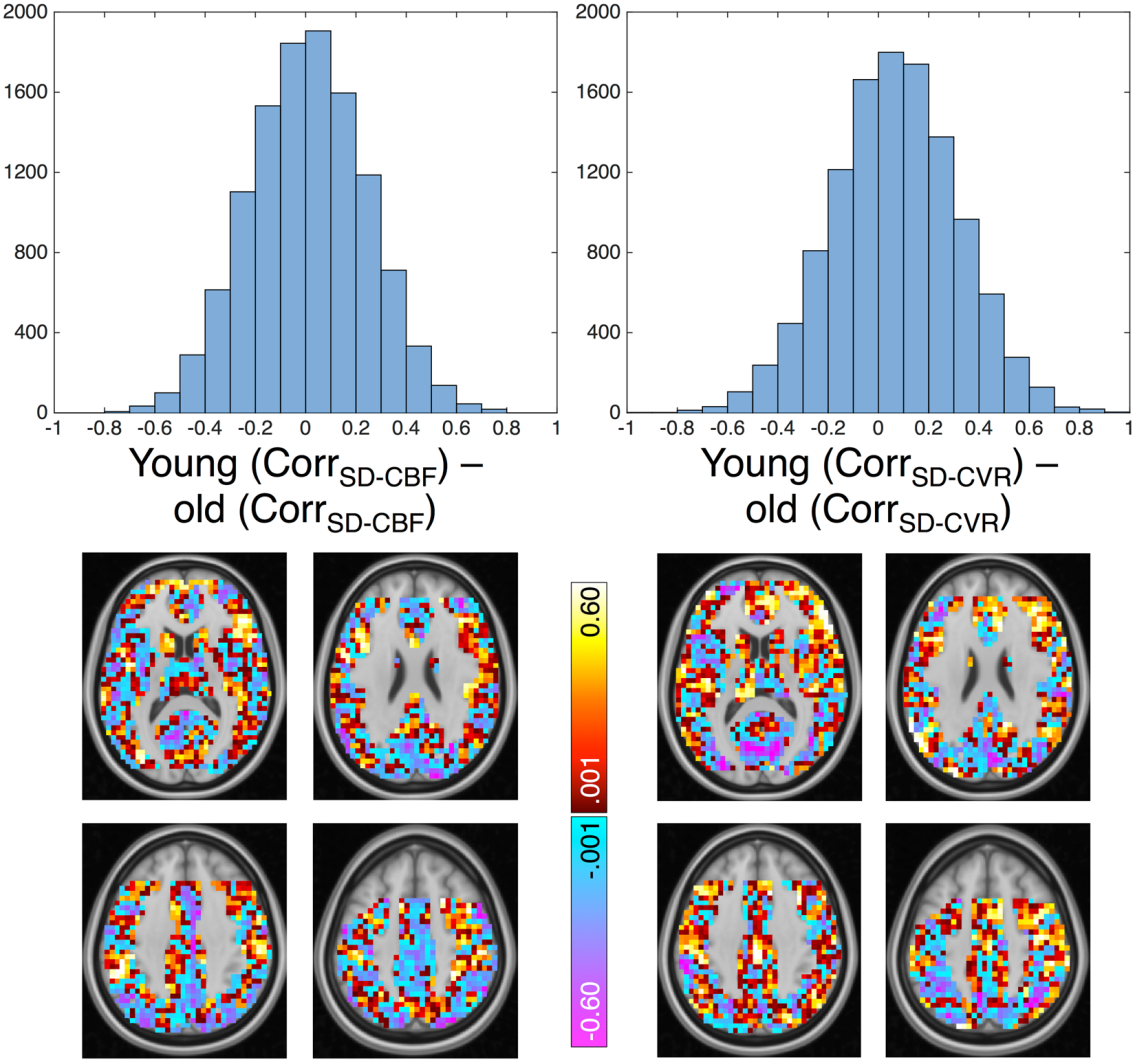
Differential voxel-wise coupling strength between SD_BOLD_, and CBF and CVR in younger and older adults. Note: Correlations between SD_BOLD_-CBF, and SD_BOLD_-CVR are computed for each voxel, across age group members. Voxel histograms represent a young minus old group difference of these voxel-wise correlation values (upper row), which are then plotted in the brain maps below each histogram (lower row). Slices for each distribution represent Z = 12 (upper left), 24 (upper right), 36 (lower left), and 48 (lower right). Orientation of images: right is right. Missing data in frontal regions are a result of partial coverage and angulation restrictions for adequate tagging in pCASL data. For complete description of both histrograms and spatial representation, these maps are intentionally unthresholded.

An alternative approach to visualizing differential coupling between SD_BOLD_fix_, CBF, and CVR can occur at the voxel (rather than subject) level, in which voxel correlations are computed across age group members. One can then visualize whether SD_BOLD_-vascular coupling differs by age group in each voxel. Raw age group differences (young minus old) in coupling are plotted in Fig. 4. Here, one can see that there are profound regional differences in whether younger or older adults show the tightest coupling between SD_BOLD_fix_ and vascular factors. Despite a modest group difference at the whole brain level (Table 2, Model 6), voxel effects are much more varied in direction. Thus, whether across regions within-person and groups, or within-voxel across persons and groups, the direction and strength of coupling of SD_BOLD_fix_ and vascular parameters varies greatly. At present, these findings highlight the challenges of simple voxel-wise vascular control of SD_BOLD_fix_ in the study of age differences.

## Discussion

Overall, our past work demonstrates that brain signal variability may index a more effective, flexible system, and that the aging brain may undergo a generalized reduction in dynamic range^1^. However, the veracity of these claims requires that age-related reductions in SD_BOLD_fix_ exist beyond vascular factors that may distinguish younger from older adults^12, 17, 20, 21, 32, 54, 70–72^. In the present study, using dual-echo BOLD/ASL, hypercapnia, and multivariate statistical methods, we examined whether lower BOLD cortical signal variability in older relative to younger adults could be accounted for by vascular parameters (CBF, CVR, and M) that are known to affect BOLD signal amplitude in older adults^17^. Our results demonstrated that SD_BOLD_fix_ remained significantly higher in younger adults after accounting for vascular effects, no matter which model we ran (Table 2, Models 1–4). These findings converge with past work attempting to control indirectly for physiological confounds in BOLD variability via techniques such as manual and semi-automated independent component analysis (ICA)^18^ denoising pipelines, PHYCAA+^19^, and mixed-model control of level and change in observed blood pressure and heart rate^8–10, 12^. In all cases, reported age effects were highly robust over and above such vascular controls, providing initial support for a neural basis for age differences in BOLD signal variability.

The study of age-related BOLD fluctuations have recently come under some scrutiny, including calls to utilize resting BOLD signal variability directly as a “vascular scaling factor” due to moderate correlations between exogenous physiological parameters (heart rate, blood pressure, respiration) and resting BOLD fluctuations (but in data that had not been physiologically denoised^14^). In our opinion, and based on our current results, one should not consider BOLD fluctuations directly as a logical proxy for vascular scaling, even though BOLD fluctuations correlate with vascular parameters to some extent. Doing so effectively attributes all signal fluctuations to a single source (vasculature), which has no basis for support in the literature. It is also trivial that simple exogenously measured physiological parameters should correlate with BOLD; this topic has been the target of a variety of denoising pipelines and techniques over the past 15 years^19, 73, 74^. The primary question in the present study is thus not whether BOLD fluctuations may relate to any physiological or vascular measure, but instead whether there is any meaningful information in BOLD signal variability over and above the influence of well-measured, brain region-specific vascular parameters. We have argued in past work that vascular issues are unlikely to account fully for BOLD variability effects, including various studies showing that BOLD fluctuations predict cognition separately within older adults^12^ and within younger adults^1, 48^, in which the role of vascular differences may be relatively small. However, what is necessary is the adequate treatment of the vascular/physiological components of BOLD fluctuations prior to the interpretation of such fluctuations. In the current study, we attempted to parameterize vascular effects in regions in which SD_BOLD_fix_ was higher in younger adults, and investigate whether we could eliminate these age differences in BOLD signal variability via vascular control. Our results demonstrate that higher BOLD signal variability in younger adults cannot be fully accounted for by individual differences in CBF, BOLD CVR, or BOLD dynamic range (M), thus further supporting the principled examination of age differences in BOLD fluctuations^1, 12, 13^.

### Seeking the most effective method(s) of vascular “control”

The most flexible form of vascular control when examining BOLD signal variability data would be to perform it at the single voxel level, which could then occur prior to and irrespective of any model of choice (like any preprocessing step). However, there are several issues with this approach at present, and we are not yet at the point where a specific correction strategy can be recommended. First, voxel-wise vascular control of BOLD parameters presumes a predictable relation between vascular measures and BOLD, within and across regions and subjects. In the present study, the correlations between SD_BOLD_fix_ and CBF, and between SD_BOLD_fix_ and CVR, varied greatly across subjects, with some participants showing very weak (near zero) correlation values, and others showing very strong relations (see Fig. 3). This result converges with recent work on young adults also suggesting inconsistent within-subject spatial relations between BOLD RSFA/ALFF and CVR^75, 76^. Although the reasons for such subject-wise variation are not yet clear, the lack of robust SD_BOLD_fix_-vascular relations in all subjects guarantees that voxel-wise scaling will have differing and unpredictable effects across subjects and brain regions. Similarly, when examining age group differences in across-subject correlations of SD_BOLD_fix_-CBF and SD_BOLD_fix_-CVR for each voxel (Fig. 4), these correlations also varied greatly in strength and direction. This result highlights that while there are many brain regions in which older adults express greater SD_BOLD_fix_-vascular coupling than in younger adults, this pattern is not consistent and provides a source of relatively unpredictable variation when enacting vascular “control” in aging studies of BOLD dynamics. Combined, these findings argue against any simple utilization of voxel-wise vascular control of SD_BOLD_fix_. Furthermore, as the BOLD signal arises from a combination of CBF, blood volume and oxidative metabolism, correction by a single component of the BOLD signal may not offer adequate correction for complex age-related vascular/metabolic differences. However, future work could pursue the nature of these very interesting regional differences in SD_BOLD_fix_-vascular relations. Improved measurement of the voxel-wise M parameter would allow this type of correction, but given the non-linear combination of low SNR measurements required, this was not possible with the current data.

One straightforward and often utilized way to improve the reliability of potentially noisy vascular estimates is to extract average CBF, CVR, and M values from multi-voxel ROIs^17, 21, 77, 78^, and either perform voxelwise or ROI-based correction of BOLD with these average values, or covary the effects of vascular parameters from effects of interest. This approached has been used in the past with task-based fMRI to show a reduced age-related difference in brain activity^21^. In the present study, we took this voxel-averaged model covariation approach, extracting average CBF, CVR, and M values from those thresholded voxels that expressed higher SD_BOLD_fix_ in younger adults. Covariation also seems a more appropriate statistical control because the effect of potentially unpredictable or unreliable vascular covariates would be handled accordingly at the model level, rather than scaled at the voxel level. We further performed log transformation (to achieve a Gaussian distributional form) and uni- and multivariate outlier detection for each vascular variable. Combined, voxel averaging and data cleaning were intended to give each vascular parameter its best opportunity to account for age-related variance in SD_BOLD_fix_ (see Table 2); importantly however, robust age differences in SD_BOLD_fix_ remained in all models we ran. Overall, future studies could use the current approach to take into account age-related vascular effects in studies of BOLD variability. While it might be unrealistic to obtain all three parameters in most contexts, BOLD CVR may be the most accessible and useful covariate given the present results. However, high quality calibrated fMRI studies would benefit most from using the M parameter, as this parameter determines the BOLD signal amplitude and is therefore theoretically the optimal parameter to best account for vascular effects.

### Potential caveats and future directions

There are important caveats to the current study that can be minimized in future work. First, the older group used here is healthier than groups typically recruited in aging studies due to the comprehensive list of exclusion criteria. Participants with controlled high blood pressure and high cholesterol were excluded from this study to prevent medication-related impacts on vascular measures such as CBF and CVR. Because of this however, the results presented likely represent a lower bound on the importance of vascular parameters on SD_BOLD_fix_. Less healthy cohorts could show a larger reduction of SD_BOLD_fix_ after accounting for vascular parameters. Second, our finding that vascular factors cannot fully account for age differences in SD_BOLD_fix_ demonstrated the impact of vascular effects in already comprehensively denoised data. It is thus possible that the impact of vascular parameters on attenuating age differences in SD_BOLD_fix_ may have increased if our data were not already denoised. However, we wanted to test our primary research questions using a preprocessing pipeline typical of our past work in BOLD variability, which argued for careful denoising to optimize age and cognition-based effects^1, 8, 9, 12^. It is also far simpler to acquire standard fMRI data and denoise them than it is to also acquire gas inhalation-based hypercapnia dataset for any study of interest. In the current study, we did both.

Third, the post-label delay utilized in the present study (900 ms) is not optimal for older populations with slower blood flow, potentially leading to an underestimation of baseline CBF and possible errors in M estimation. These factors may be minimized in the present sample given their above-average health status. This may however contribute to differential relationships between CBF, M and SD_BOLD_fix_ (it would not, however, have an impact on the relationship between CVR and SD_BOLD_fix_). Future studies could use multiple delay pCASL data to yield more accurate CBF and M estimates. While BOLD CVR is a sensitive marker of vascular aging, the M parameter is in theory the most relevant parameter for investigating the impact of declining vascular health on the amplitude of BOLD signal fluctuations (since it represents explicitly the BOLD dynamic range). Future studies could also use more recent BOLD calibration models^45, 68, 79^ to investigate these effects further by not only allowing measurement of vascular effects in BOLD, but also allowing one to account for the impact of resting metabolism. Such models help to better determine dynamic range given that BOLD signal amplitude depends partly on how much deoxyhemoglobin is present at rest.

Fourth, many papers examining vascular effects in BOLD fluctuation amplitudes have utilized resting-state data^14, 33, 34^, whereas we have examined fixation block data as a proxy for resting state. Although it has been argued that fixation block data (from a broader block design study) may not be a valid proxy for resting state given that external cognitive engagement may modify resting activity^80–82^, there has been no evidence to date that such an effect exists for BOLD variability measures; in fact, ongoing work in our group suggests that pre- and post-task resting-state BOLD variability, and fixation block-based BOLD variability, are all very highly correlated and show very similar spatial patterns in relation to aging (Grady and Garrett, *under review*). Finally, future studies could examine the extent to which age- and cognition-related differences in artifact-free BOLD signal variability (in particular, independent of hypercapnia-based vascular effects) correlates with artifact-free EEG/MEG signal variability (e.g., power, entropy, dimensionality). This would permit a more comprehensive assessment of the neural basis of age- and cognition-related differences in brain signal dynamics^14^. Interestingly however, increasing work on the vascular basis of EEG/MEG suggests that hypercapnia may impact some aspects of electrical brain signals and their associated brain states in healthy young adults^83, 84^. Thus, future multi-modal assessment of brain dynamics may benefit from covarying hypercapnia-based vascular effects from both BOLD and electrical signals prior to examining age differences in brain dynamics to avoid biasing model results.

## Conclusion

Overall, our results demonstrated that brain regions in which younger adults expressed higher SD_BOLD_fix_ remained robust after accounting for vascular effects^1, 12, 13^. Our findings thus further establish and promote the principled study of aging-related BOLD dynamics.
